# Routine vitamin A supplementation and other high impact interventions in Sierra Leone

**DOI:** 10.1111/mcn.13041

**Published:** 2020-07-27

**Authors:** Aminata S. Koroma, Sulaiman G. Conteh, Mariama Bah, Habib I. Kamara, Mohamed Turay, Abdulai Kandeh, Anna Macauley, Henry Allieu, Anita A. Kargbo, Mustapha Sonnie, Mary H. Hodges

**Affiliations:** ^1^ Food and Nutrition Ministry of Health and Sanitation Freetown Sierra Leone; ^2^ Reproductive Health and Family Planning Program Ministry of Health and Sanitation Sierra Leone; ^3^ Nutrition Helen Keller International Freetown Sierra Leone

**Keywords:** child survival interventions, complementary feeding, deworming, family planning, immunization, maternal health, Sierra Leone, teenage, vitamin A supplementation

## Abstract

In 2017, transition to routine vitamin A supplementation (VAS) commenced as an integrated reproductive and child health service including vaccinations, Albendazole for deworming, complementary feeding demonstrations, ‘quality’ family planning counselling and provision of modern contraceptives. After 10 months, a lot quality assurance sampling survey evaluated coverage of these interventions. Each of three districts was divided into five supervision areas (lots), and 19 villages were randomly selected in each lot proportional to population size. Households were randomly selected, and a questionnaire was administered to a caregiver of a child 6–11, 12–23 and 24–59 months in each village. Overall, caregivers of 855 children were interviewed, and 19 questionnaires were completed for each age group (6–11, 12–23 and 24–59 months) in each of the five lots in each district. All lots in one district passed the threshold of 80% for VAS and 75% coverage for Albendazole, and two lots failed for either VAS/Albendazole in the other two districts. Overall, weighted VAS coverage for children 6–59 months was 86.9%, and weighted Albendazole coverage for children 12–59 months was 80.9%. Most caregivers (77.2%) knew that complementary feeding should be introduced at 6 months, 44.9% were providing three or more (of six) food groups, 84.9% were aware of family planning and 37.5% were using a modern contraceptive. Integration of reproductive and child health services appears to be a suitable platform for routine VAS and Albendazole whilst improving complementary feeding practices and access to family planning.

**Key messages**

- The lot quality assurance sampling survey found the integration of reproductive and child health services at a routine six monthly contact points to be a suitable platform for the transition of mass vitamin A supplementation into routine services.
- Overall, by caregivers' recall, vitamin A supplementation and Albendazole coverage was greater than 80% and 75%, respectively, 77% of caregivers knew that complementary feeding should be introduced at 6 months, 45% recalled having provided three or more (of six) food groups over the last 24 h, 85% were aware of family planning and 38% were using a modern contraceptive.

## 1 INTRODUCTION

Sierra Leone has the highest child under 5 years mortality rate, (World Health Organization [WHO], 2016) the highest reported maternal mortality ratio and the highest lifetime risk for women dying in childbirth worldwide (Sierra Leone Reproductive Maternal Newborn and Child Strategy, 2017‐2021). The Ebola emergency in Sierra Leone 2014–2015 had a devastating impact on the health sector including access to and utilization of reproductive and child services (Sierra Leone Food assistance fact sheet 2017).

From 2006 to 2017, Sierra Leone held mass campaigns during biannual maternal and child health weeks to achieve universal coverage of high‐impact and cost‐effective maternal and child survival interventions. These interventions include vitamin A supplementation (VAS), Albendazole for deworming and vaccines such as polio and measles. The mass campaigns achieved effective, equitable coverage of VAS, Albendazole and vaccinations by district, age group, mothers' religion and occupation (Sesay et al., 2015).

Mass campaigns are expensive, donor driven and highly reliant upon funding for mass vaccination campaigns. They require extensive macro planning at the national and international level for timely ordering, shipment and distribution of commodities and micro planning at all levels: national, district and at every peripheral health unit (PHU). Campaigns do not encourage health‐seeking behaviours within vulnerable communities or build the capacity/resilience of routine services to respond to that demand. Children may also receive services later than indicated on their health cards, because the campaigns only take place every 6 months. For example, an infant who is 5 months of age during a campaign would receive their first VAS at 11 months rather than at 6 months when it is recommended. After 6 months of age, breastmilk needs to be complemented by other foods to meet the child's nutritional needs for rapid growth. Appropriate complementary feeding provides key nutrients (e.g., micronutrients, essential fatty acids, protein and energy). Inadequate complementary feeding can restrict growth and jeopardize child survival and development (©United Nations Children's Fund, 2011).

In 2010, the primary donor funding VAS, the Canadian International Development Agency, requested Helen Keller International (HKI) develop a platform that could achieve effective VAS coverage at 6 months of age in preparation for transitioning from mass to routine VAS (rVAS), as it was anticipated that funding for polio campaigns would decline as polio‐elimination goals were met in the region. In 2011–2012, a 6‐month contact point (6MCP) was piloted that included rVAS, infant and young child nutrition (IYCN) counselling with mother's participation in the preparation of complementary food made from a preroasted blend of locally available ingredients and confidential family planning (FP) counselling and provision of long‐term hormonal implants (Jadelle), which are the most commonly used long acting reversible contraceptives methods as few health workers have been trained in intrauterine contraceptive devices (IUCDs).

An evaluation of the pilot found that at 6–7 months of age, 75% of infants had received rVAS, 96% had been fully vaccinated, 64% of mothers had participated in the preparation of complementary food and 75% had been counselled on FP with 45% (Hodges et al., 2015). During the Ebola emergency, health facilities offering the integrated package of services at the 6MCP were able to retain significantly higher health‐seeking attendance than those that had not integrated and were able to offer rVAS and vaccination when a mother attended for participatory IYCN and quality confidential FP services (Conteh et al., 2016).

By mid‐2017, the 6MCP had been integrated into 340 (of 1,280) (PHUs nationwide and national overall rVAS coverage for children 6 to 59 months had increased from under 10% to approximately 35% (HMIS reports 2018). In the catchment communities reached by the 6MCP, complementary feeding practices for children 6–23 months of age had improved significantly compared with the previous national surveys: minimal meal frequency (58%), minimal dietary diversity (49%) and consumption of vitamin A‐rich foods (17%). In addition, 97% of mothers were aware of FP and 53% were taking modern contraceptives (Koroma et al., 2019). In early 2018, the full transition to rVAS and Albendazole commenced in three districts (Bo, Kenema and Koinadugu), and mass VAS and Albendazole during mother and child health weeks ceased. Services were delivered at the facility level, with outreach sessions organized by PHU staff for remote communities in their catchment area. The district health management teams (DHMTs) and HKI worked with all PHUs to reconfirm the remote communities in their catchment area and developed a schedule of monthly outreach child services for immunization, VAS and deworming with Albendazole. Monthly in‐charges meeting held by the DHMTs were regularly attended by the HKI‐focal person to report supervisory findings and reinforce best practices. These meetings were held every 2 months in Koinadugu due to the longer distances involved and time required. After 10 months, a lot quality assurance sampling survey (LQAS) was conducted to evaluate the coverage of these interventions and is presented in this manuscript.

## 2 METHODS

A LQAS survey was conducted to ascertain whether performance thresholds of 80% for VAS and 75% for Albendazole had been reached in each lot and to estimate coverage for VAS, Albendazole, complementary feeding and FP practices. The availability of essential commodities (vitamin A, Albendazole, male condoms, oral contraceptive pills, Depo‐Provera and hormonal implants), and the training status of health workers and community health workers (CHWs) was also evaluated. The LQAS survey was conducted from September 27 to October 1, 2018, in Bo and Kenema districts, and from October 8 to 12 in Koinadugu district.

**FIGURE 1:**
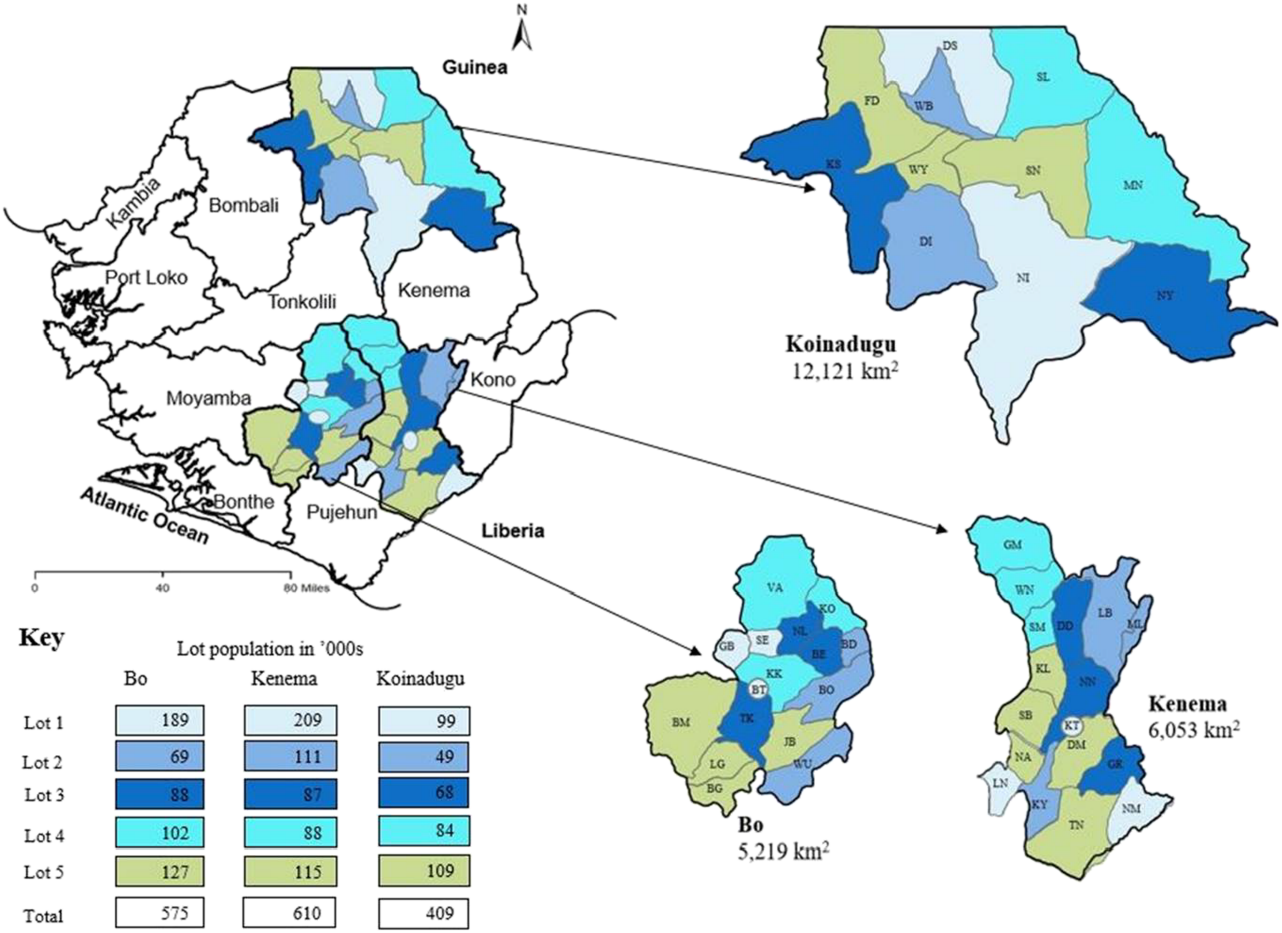
Map of districts, chiefdoms and lots

A total of 285 surveys were completed in each district. Nineteen surveys were completed for each age group (6–11, 12–23 and 24–59 months) in each of the five lots in each district. The population size of each lot and district is shown in Figure 1.

### 2.1 Questionnaire development

Households were randomly selected, and questionnaires were pretested to determine suitability, sequencing of questions and amount of needed time per interview. Both English and Krio versions of the surveys were then programmed into Open Data Kit, uploaded to a web‐based platform, Ona.io., then downloaded onto Samsung tablets. Three slightly different questionnaires were developed for mothers/caregivers of infants aged 6–11 months, mothers/caregivers of children aged 12–23 months and mothers/caregivers of children aged 24–59 months as summarized in Table 1 and available in full in Data S1.

**TABLE 1:** Questionnaire summary

Sample group	Indicators measured
Caregiver of children 6–11 months of age	Receipt of vitamin A supplementation (VAS) •Reported that their child had received a dose of vitamin A in the last 6 months and/or confirmed by inspection of the child health card (when available) •Had knowledge about VAS (where to go, when to go and how frequently) Complementary feeding knowledge and practices •Had knowledge that complementary feeding should be commenced at 6 months •Had feed at least 3 (of 6) food groups to their infant in the last 24 h Family planning knowledge and practices •Were aware of family planning (FP) methods •Were practicing modern or tradition contraception or abstinence
Caregiver of children 12–23 months of age	VAS as above •Recalled their child had received Albendazole (ALB) in the last 6 months and/or confirmed by inspection of the child health card •Recalled their child had received Pentavalent 3 and proportion and/or confirmed by inspection of the child health card
Caregiver of children 24–59 months of age	VAS as above Deworming as above
Health worker (HWs) in the local PHU	•Had been train on the 6 monthly contact point •Had cascaded trainings to others •Had attended the 8 days training on ‘quality’ FP counselling and the insertion and removal of hormonal implant •Had training materials available at the PHU •Had stock outs of vitamin A, ALB, and modern contraceptives at the time of LQAS •Had trained and retained community health workers

Abbreviations: LQAS, lot quality assurance sampling survey; PHU, peripheral health unit.

### 2.2 Training

In October 2018, 38 enumerators (undergraduate/postgraduate students) with local language skills and experience in mobile data collection were trained on the LQAS survey, digital data collection and interviewing. One training was held in Bo Town for enumerators who would be administering the survey in Bo and Kenema districts. A second training was held in Kabala, for enumerators who would be administering the survey in Koinadugu district. A posttest was administered, and the 30 enumerators with the best performance on the posttest were retained to conduct the survey (10 per district). Trainings consisted of 1 day in the classroom, 1 day of field practice and 1 day for debriefing. Each pair of interviewers received a tablet to enter data and hard copies of the questionnaires.

### 2.3 Village protocol and household selection

Nineteen villages were randomly selected to be surveyed in each lot. The villages to be surveyed were determined using probability proportional to size sampling, (LQAS participant's manual, USAID, http://polioeradication.org/wp‐content/uploads/2016/09/Assessing‐Vaccination‐Coverage‐Levels‐Using‐Clustered‐LQAS_Apr2012_EN.pdf). Upon arrival at each village, the enumerators introduced themselves to the local head man and explained the purpose of their visit, asking for one ‘helper’ to map the village and estimate the number of houses. After the map of each village was drawn, the village was divided into four sectors of similar population size using landmarks (e.g., roads, rivers, schools, mosques and churches). One sector was randomly selected by ballot, and each house was numbered with chalk. If that selected sector had less than 20 households, enumerators randomly selected one household using a random number table and used it as their starting point.

### 2.4 Identifying respondents

At the selected household, the enumerators introduced themselves, explained the purpose of their visit and requested consent to proceed. If no eligible children resided in the household, the next household on the left was selected. If a household had more than one child in an age group, the names of the children were written down on small pieces of paper, one was selected at random, and the caregiver of that child was interviewed. If children of different age groups resided in the household, one survey was administered for each age group.

### 2.5 Survey administration

Children's ages were verified with health cards whenever possible. When health cards were unavailable, children's ages were estimated using a calendar of events. Vitamin A capsules (red and blue) and Albendazole tablets were shown by enumerators to assist caregivers with recall The child health card was also requested and inspected to assess whether VAS or Albendazole had been recorded by the health worker. Interviewers recorded data directly into the tablets using the Ona application.

### 2.6 Supportive supervision

Supportive supervision was conducted during the first 2 days of the 5‐day survey. The training teams, which comprised representatives from the Directorate of Food and Nutrition, MoHS and HKI, supervised the enumerators in their use of the survey protocol, village identification, mapping, segmentation, random sampling and interviewing technique. The Ona account administrator crosschecked the number of interviews performed, the GPS location and the time taken to conduct interviews. Some data fields were mandatory and programmed to alert for errors.

### 2.7 Health workers questionnaire

Staff from at least five PHUs were interviewed from each lot to evaluate the level of training, stock out of commodities and number of trained CHWs who were actively working for the PHU.

### 2.8 Debriefing review meetings with the enumerators, HKI and the DHMTs

A day after the survey work, a 1‐day debrief meeting was held with the interviewers and members of the DHMTs, the Directorate of Food and Nutrition and HKI to report on the results and discuss challenges met during the transition from mass to rVAS and during the survey.

### 2.9 Data analysis and sample size calculation

A sample size of 285 provides a point coverage estimate with a ±10% confidence interval at the district level for each age group. Data analysis was done using Microsoft Excel 2010 and SPSS version 21. For key indicators, the statistical precision was assessed using 95% confidence intervals. District‐level coverage was calculated using an Excel template, which weighted the lot results based on lot population to achieve representative estimates (WHO, 2017). Chi squared tests were used to compare groups. If a child health card was not available, VAS and Albendazole were calculated using the number of cards seen as the denominator.

### 2.10 Ethical considerations

The Directorate of Food and Nutrition submitted the survey protocol to the Sierra Leone Ethics and Scientific review committee for approval. Having conducted a review of the study protocol and determined its minimal risk, the committee granted an approval for the study. A letter from the Chief Medical Officer was sent to District Medical Officers and other key officials to introduce the survey teams and request their cooperation. Copies were made available to the interviewers to enable cooperation at village level. Informed verbal consent (translated into the local language) were obtained before any individual was interviewed.

## 3 RESULTS

### 3.1 Cohort characteristics

Nineteen interviews for each of three age groups were completed in each lot for a total of 855 interviews (three districts of 285 interviews each). The cohort characteristics are shown in Table 2.

**TABLE 2:** Cohort characteristics

Indicators	Bo (*N* = 285)		Kenema (*N* = 285)		Koinadugu (*N* = 285)	
	*n*	%	*n*	%	*n*	%
**Sex of child**						
Male	125	43.8	142	49.8	136	47.7
Female	160	56.1	143	50.2	149	52.3
**Marital status and age of caregivers**						
Married	241	84.6	253	88.8	276	96.8
Single	44	15.4	32	11.2	9	3.2
**Religion of caregivers**						
Muslim	217	76.1^a^	251	88.1	248	87.0^a^
Christian	68	23.9	34	11.9	37	13.0
**Education status of caregivers**						
None	120	42.1^b^	166	58.2	187	65.6^b^
Primary	88	30.8	2	0.7	43	15.1
Secondary	71	24.9	44	15.4	48	16.8
Tertiary	5	1.7	71	24.9	3	1.3
Other (vocational, Islamic)	1	0.4	2	0.7	4	1.4
**Occupation status of caregivers**						
Farmer	153	53.7^c^	173	60.7	207	72.6^c^
Trader/business	65	22.8	72	25.3	52	18.2
Unemployed/stay home	44	15.4	14	4.9	10	3.5
Other	23	9.0	26	10.0	16	5.6
**Relationship of caregiver to child**						
Mother	256	89.8	259	90.8	276	96.8
Grandparent	12	4.2	12	4.2	3	1.0
Father, aunt/other	8, 9	6.0	6, 8	4.9	5, 1	2.1

^a^ Significant differences between Bo versus Koinadugu for religion, (*p* < 0.05),

^b^ Significant differences between Bo versus Koinadugu for no education (*p* < 0.01),

^c^ Significant differences between Bo versus Koinadugu for farmers (*p* < 0.001).

The only significant differences in cohort characteristic by district were between Bo versus Koinadugu for religion, occupation and educational status (*p* < 0.05, <0.001, <0.01, respectively).

### 3.2 Routine vitamin A, Albendazole and Pentavalent 3 coverage

Across all districts, weighted VAS coverage among children 6–59 months based on verbal affirmation was 86.9% (Bo: 92.3%, Kenema: 86.1% and Koinadugu: 81.7%) and based on card confirmation was 71.9%, (81.4%, 69.2%, and 47.7%, respectively) (Table 3). There was significantly higher VAS coverage in Bo versus Kenema and Koinadugu districts (*p* < 0.01, <0.0001 respectively). VAS coverage by verbal affirmation and card confirmation was lower in 12–23 and 24–59 months versus 6–11 months in all three districts, although none of these were statistically significant (Table 3).

**TABLE 3:** VAS, ALB and Pentavalent 3 weighted coverage by district and by age group

Age group (months)	Bo		Kenema		Koinadugu		
	*n*	% (95% CI)	*n*	% (95% CI)	*n*	% (95% CI)	
Verbal affirmation							
VAS 6–59	264	92.3 [91.2, 94.0]	248	86.1 [86.7, 87.3]	231	81.7 [80.8, 81.4]	
ALB 12–59	169	90.8 [87.8, 90.0]	147	76.7 [76.2,78.6]	145	77.2 [75.1, 77.4]	
Penta 3 12–23	89	94.6 [90.0, 99.2]	78	82.4 [72.8, 92.0]	80	83.9 [76.3, 91.5]	
Card confirmation							
VAS 6–59	215/264	81.4 [79.0, 83.8]	189/273	69.2 [66.5, 72.0]	127/266	47.7 [44.7, 50.8]	
ALB 12–59	109/175	62.3 [58.7, 65.9]	107/179	59.8 [56.1,63.4]	69/180	38.3 [34.7, 41.9]	
Penta 3 12–23	80/98	81.6 [77.7, 85.6]	62/90	68.9 [64.1, 73.7]	61/92	66.3 [61.5, 71.2]	
	6–11 months		12–23 months		24–59 months		Overall
Verbal affirmation							
VAS	240	84.2 [81.8, 86.9]	256	89.8 [88.1, 92.2]	247	86.7 [85.4, 88.3]	86.9 [86.3, 88.0]
ALB			218	76.5 [74.8, 78.9]	243	85.3 [83.9, 86.9]	80.9 [80.5, 81.4]
Penta 3			247	86.7 [84.9, 89.1]			86.7 [84.9, 89.1]
Card confirmation							
VAS	183/272	67.3 [64.4, 70.1]	197/271	72.7 [70.0, 75.4]	197/260	75.8 [73.1, 78.4]	71.9 [70.3, 73.4]
ALB			148/270	54.8 [51.8, 57.8]	137/264	51.9 [48.8, 54.9]	53.4 [51.2, 55.5]
Penta 3			203/280	72.5 [69.8, 75.2]			72.5 [69.8, 75.2]

Abbreviations: ALB, Albendazole; CI, confidence interval; VAS, vitamin A supplementation.

In all districts, weighted Albendazole coverage among children 12–59 months based on verbal affirmation was 80.9% (Bo: 90.8%, Kenema: 76.7%, and Koinadugu: 77.2%), and based on card confirmation was 53.4% (Bo: 62.3%, Kenema: 59.8%, Koinadugu; 38.3%) (Table 3). There was significantly lower coverage based on card confirmation in Koinadugu versus the other districts (*p* < 0.01 each).

Weighted district‐level coverage of Pentavalent 3 among children 12–23 months based on verbal affirmation was 86.7% and by card confirmation was 72.5%. There was no significant difference in Pentavalent 3 coverage between districts (Table 3). There were no significant differences in VAS, Albendazole or Pentavalent 3 coverage by the child's sex, mothers' religion, educational status or occupation.

**FIGURE 2:**
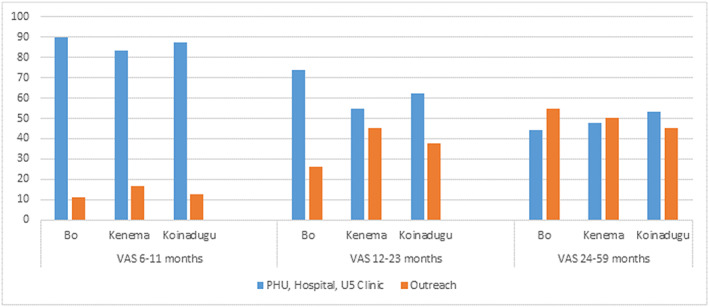
Venue of vitamin A supplementation (VAS) receipt by district and by age group. Overall, significantly more caregivers of children 12–23 and 24–59 months reported their child had received VAS from outreach services than children 6–11 months (*p* < 0.001, *p* < 0.0001, respectively)

**FIGURE 3:**
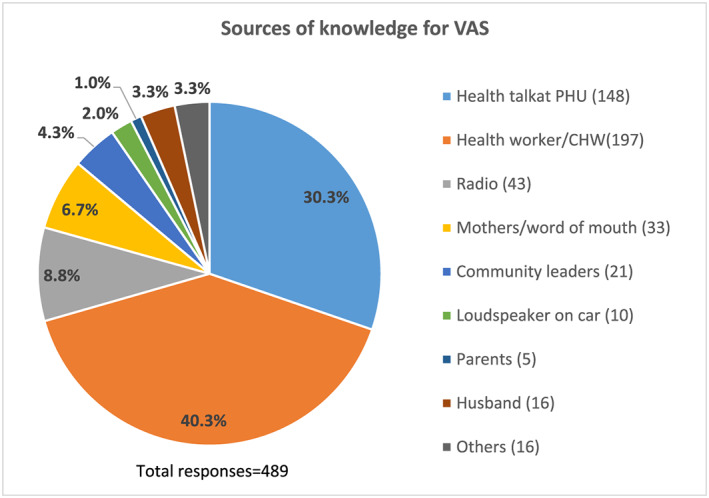
Sources of knowledge for vitamin A supplementation (VAS). CHW, community health worker; PHU, peripheral health unit

Caregivers of children age 6–11 months were significantly more likely to report that their child received VAS from a PHU compared with caregivers of children age 12–23 and 24–59 months (*p* < 0.001 and 0.0001 respectively). Caregivers of children 12–23 months and 24–59 months were more likely to report their child received VAS from outreach services (Figure 2). Most caregivers had heard about VAS from health workers/CHWs (40.3%) or from a health talk at the PHU (30.3%) (Figure 3).

### 3.3 Complementary feeding practices by caregivers of children 6–11 months old

**FIGURE 4:**
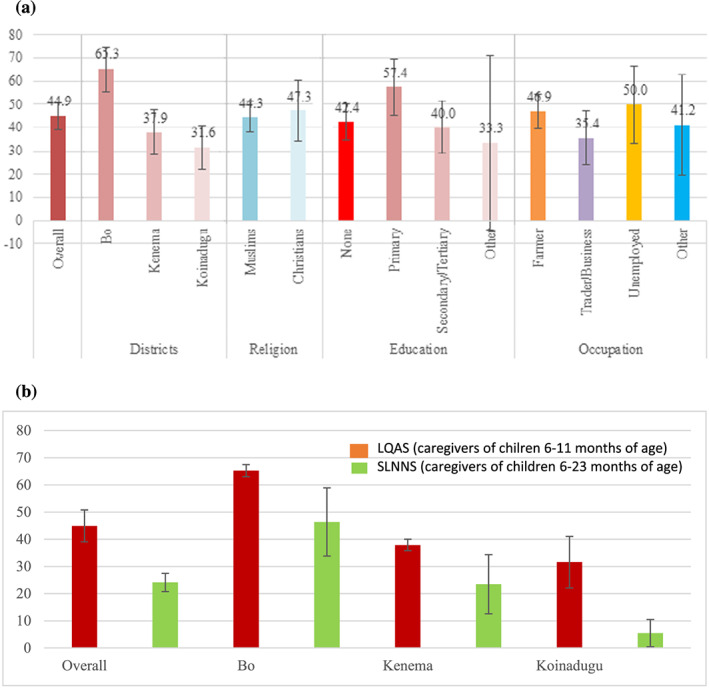
(a) Mean minimum dietary diversity reported by 285 caregivers of infants 6–11 months by district and caregivers' religion, education and occupation (95% confidence interval [CI]). (b) Mean minimum dietary diversity reported by caregivers of infants 6–11 months in the lot quality assurance sampling survey (LQAS) (2018) and by caregivers of children 6–23 months in the Sierra Leone National Nutrition Survey (SLNNS, MoHS, 2017) (95% CI)

Approximately three quarters, (77.2%, 95% CI [74.3.2, 79.7]) of caregivers knew that complementary foods should be introduced at 6 months, Bo: 83.2% (95% CI [81.2, 85.2]), Kenema: 90.5% (95% CI [90.1, 91.0]) and Koinadugu: 57.9% (95% CI [56.8, 59.2]). Overall, 44.9% (95% CI [36.9, 46.3]) of caregivers were providing three or more (of six) food groups, in addition to breast milk, as shown in Figure 4a. Significantly, more caregivers were providing three or more (of six) food groups in Bo: 65.3%, (95% CI [64.1, 66.3]) versus Kenema: 37.9% (95% CI [36.9, 39.0]) and Koinadugu: 31.6% (95% CI [22.17, 40.98]) (*p* < 0.01, <0.001 respectively). There were no significant differences in the provision of three or more food groups by mothers' religion, education or occupation (Figure 4a).

Significantly, more caregivers were providing complementary feeding with three or more food groups compared with the Sierra Leone National Nutrition Survey (SLNNS, MoHS, 2017) in each district: Bo 65.3% versus 46.3%, Kenema 37.9% versus 23.5% (*p* < 0.05 each) and in Koinadugu 31.6% versus 5.5% (*p* < 0.0001each) as shown in Figure 4b.

### 3.4 Sources of knowledge, of FP and uptake among caregivers of children 6–11 months old

Overall, 84.9% (95% CI [83.2, 85.4]) of caregivers had heard about FP, significantly more in Bo (96.8%) versus Koinadugu (73.7%) (*p* < 0.001) (Table 4). The main sources of information on FP were health workers and hospitals/PHUs. Overall, 37.5% of caregivers were using modern contraceptives equitable by caregivers' education level, religion and occupation but higher in Bo (47.4%) and Kenema (44.2%) versus Koinadugu (21.1%) (*p* < 0.05 each). The most common modern contraceptives known by caregivers were depo, pills and hormonal implants (known locally as captain band, Table 4). These were also the most commonly used commodities.

**TABLE 4:** Sources of knowledge, on FP and uptake among 285 caregivers of children 6–11 months old

	Overall (*N* = 285)	
	*n*	%
**Heard about FP**	242	84.9
**Sources of knowledge of modern contraceptive**		
Health worker	146	51.2
Hospital/PHU	138	48.4
Friend	26	9.1
Radio	27	9.5
Mother support group	16	5.6
Other (Marie Stopes, husband, herbalist, school)	29	10.2
**Knowledge of commodities among caregivers of children 6–11 months old**		
**Commodity**	*n*	%
‘Captain band’ (hormonal implant)	209	73.3
Pills	193	67.7
Depo	182	63.9
Country rope/herbs	52	18.2
IUD/coil	44	15.4
Condoms	36	12.6
**Contraceptives used among caregivers of children 6–11 months old**		
Not using	178	62.5
Depo	42	14.7
Pills	29	10.2
Hormonal implant	20	7.0
Abstinence	10	3.5
Condoms	3	1.1
Country rope/herbs	3	1.1

Abbreviations: FP, family planning; IUD, intrauterine device; PHU, peripheral health unit.

### 3.5 Health worker interviews to ascertain training and stock‐out status

A total of 106 health workers ‘in charge’ were interviewed. Overall, 53.8% had been trained on the 6MCP; 72.6% had cascaded their training to other staff at their PHU; 68.9% still had training materials on site; 87.7% had received the 8‐day training on FP counselling and the insertion of hormonal Jadelle implants; 91.5% had trained CHWs and 88.4% had be able to retain CHWs. The lowest CHWs retention (58.9%) was in Kenema. Overall, at the time of the visit stock outs of vitamin A 100,000 IU was 8.5%, vitamin A 200,000 IU: 7.5%, Albendazole: 17.9%, oral contraceptive pills: 10.4%, Jadelle implants: 21.7%, condoms: 44.3% and Depo‐Provera: 54.7%.

## 4 DISCUSSION

The 6‐monthly contact point at PHUs augmented by outreach services demonstrated effective rVAS and Albendazole weighted coverage by verbal affirmation in all three districts despite their inherent differences in demography and the resources available. Confirmation by inspection for the child health card was consistently lower in all districts perhaps due to caretakers having overestimated coverage or health workers having forgotten to mark the card. The programme is now being taken to scale over the next 4 years as mass campaigns for VAS are phased out.

In general, Bo district performed better than Kenema district and both did better than Koinadugu district with higher coverages of VAS, Albendazole and Pentavalent 3 by verbal affirmation and card confirmation. The population in Bo is generally more educated and has the highest ratio of PHUs per capita:132 PHUs serving a population of 575,478 in an area of 5,219 km^2^ (Statistics Sierra Leone, 2015; Statoids, 2017). Kenema has fewer PHUs (123) but serves a larger population (609,891) spread across a larger area: 6,053 km^2^. Koinadugu has only 75 PHUs serving a population of 409,372 in the largest area: 12,121 km^2^. This gives an average population per PHU of 4,360, 4,958 and 5,458 and catchment areas of 40, 49 and 162 km^2^ per PHU, respectively. The introduction of paid CHWs in 2018 should help alleviate the challenge of distances to PHUs (Ministry of Health and Sanitation, 2016), but fewer CHWs had been retained in Kenema by the time of this LQAS due to challenges in the distribution of their stipends.

At the debrief meetings with the DHMTs, the long distances to some PHUs, irregular outreach service provision and the cost of getting to the PHU were brought out as barriers to seeking routine health services. The DMHT, caregivers and PHU staff considered that the most influential motivation for attendance was the mothers' participation in the preparation of a complementary food and feeding her child. In communities that have to travel over 10 km to a PHU with limited access to markets, the opportunity to access an infants' meal was highly appreciated by mothers and recognized by health workers and the DHMTs. In addition, the preparation of a complementary food focuses attention and facilitates instruction on introduction of complementary feeding at 6 months of age and diversity of complementary diets.

Each PHU has a micro plan to schedule their outreach services and defaulter tracing in their remote communities. The outreaches are frequently scheduled for Fridays and Saturdays when mothers and children are more accessible due to school closures on these days in Muslim communities. Outreach services might be more effective if they were scheduled to coincide with local market days when many mothers who are trading and will be in attendance with their infants. A higher proportion of coverage was achieved by outreach in older age groups as the older age groups do not attend PHUs as often, having completed their immunization schedules.

Emphasis and funding for outreach services for communities far from the PHU and for DHMTs to provide supportive supervision needs to be maintained if effective coverage is to be sustained. In addition, more funding is required to improve the ratio of PHUs per population and per km^2^.

The DHMTs in Kenema have recommended an additional 10 buildings built/allocated by remote communities of sufficient size and characteristics but are unable to proceed due to lack of funding for staff salaries.

Monthly in‐charges meeting held by the DHMTs and regularly attended by the HKI‐focal person were identified as an opportunity to reinforce best practices on the 6MCP and help improve data collection through the health management information system (HMIS). The VAS coverage through the HMIS for the corresponding period was 51.8% in these three districts (compared with verbal affirmation of 86.7% and card confirmation of 71.9% in the LQAS). Records of VAS are based upon health workers' reports, and if the health worker does not record the service on the child health card or the ledger, the data will not flow into the HMIS.

Dietary diversity in complementary feeding for children 6–11 months of age was defined as three or more of six food groups is similar to that found in the evaluation of the 6MCP, 2017 (49.2%) and significantly higher compared with children 6–23 months of age in the SLNNS, MoHS, (2017), (29.1%). Bo had a higher proportion of mothers providing dietary diversity compared with Koinadugu, despite the finding of more farmers there. Caregivers were apparently unaware of the importance of providing a diverse diet to infants 6–11 months of age to complement breast feeding. Caregivers may have had little influence of the utilization of home‐grown foods for household consumption rather than for sale to local markets and/or to the capital as men most frequently make decision about how the produce is used and income is spent within a household.

It has previously been demonstrated that the major barriers to diversity of complementary feeding are availability, affordability and accessibility even when caregivers are aware of the advice given by health workers (Turay H et al., 2013). In the post‐Ebola context, 50% of households in Sierra Leone was found to be food insecure. With a national population growth rate of 3.2% per annum (Ministry of Health and Sanitation, 2015), this challenge may be exacerbated as access to fertile arable land is finite and families that move away from their communities of origin are less likely to have access to fertile land (Food and Agricultural Organisation, 2017). Integrating nutrition‐specific interventions such as complementary feeding and FP counselling into health systems may help ensure effective service delivery whilst having an impact on nutrition outcomes (Salam, Das, & Bhutta, 2019).

Access to modern contraception is a MoHS priority and its uptake is usually associated with female educational status and further contributes to female empowerment (Sierra Leone Demographic Health Survey, 2015). Teenage pregnancies are a major concern in Sierra Leone with 26% of girls giving birth before 18 years of age (Government of Sierra Leone, 2015). The rate of modern contraceptive use in this study (37.5%) was higher than the current national prevalence for all women of reproductive age (27%) and for married women (20%) and higher than the national target (30%) for 2020 (Sierra Leone Commitment Maker, 2019). However, 15.1% of respondents said they were unaware of ‘family planning’ and 1.1% were still using traditional methods such as ‘herbs or country rope’ (a fetish tied around the waist). Stock outs of modern contraceptive commodities remains high in all three districts limiting a woman's' choice.

This is reflected by their uptake and few health workers have been trained to counsel for and insert an IUCD. The next phase of scale up of the integrated package of reproductive and child health services will begin to train various cadres of health workers on the safe provision of IUCD as part of the training on FP.

This study is limited in that it did not include baseline or control from non‐6MCP catchment communities. Both this study and the SLNNS, MoHS (2017), were conducted before the ‘lean season’ when access to stored crops from the previous harvest had been depleted. Comparison regarding dietary diversity will be limited due to different sample sizes, locations and age groups, which may have introduced bias.

## 5 CONCLUSION

The integration of reproductive and child health services appears to be a suitable platform for transitioning from mass to rVAS and Albendazole whilst improving complementary feeding practices and access to FP despite the challenge of frequent commodity stock outs. District‐specific performance appears to be related to their cohort characteristics and the population density served by the PHUs.

## CONFLICTS OF INTEREST

The authors state that there is no competing interest and the contents are the responsibility of the authors and do not necessarily reflect the views of the Irish Aid, UNICEF, UNFPA or the Canadian Government. The 6MlyCP is co‐funded by Irish Aid and Global Affairs Canada. The United Nations Fund for Population Activities (UNFPA) provide FP commodities and the United Nations Children's Fund (UNICEF) provide vitamin A capsules, Albendazole, vaccines and child health cards to the MoHS. This study was made possible by the generous support of the Global Affairs Canada through UNICEF to Helen Keller International. The funders had no role in data collection and analysis, decision to publish or preparation of the manuscript.

## CONTRIBUTIONS

ASK manages the MoHS‐DFN. SGC, who served as the manager of RHFPP‐MoHS, coordinated and facilitated all FP trainings and supervisions. AM, HA and AJ supported the implementation and supervision at district level. MS and MB supervise the programme. AbK, HIK, MB, MT and MHH designed the study. MB, AM, HA and AK pretested the questionnaire. HIK, MT and AbK compiled and analysed the data. MB drafted and MH reviewed the manuscript. All authors approved the final manuscript.

## Supporting information

Data S1. Supporting Information
